# Tyrosine decarboxylase activity of enterococci grown in media with different nutritional potential: tyramine and 2-phenylethylamine accumulation and *tyrDC* gene expression

**DOI:** 10.3389/fmicb.2015.00259

**Published:** 2015-04-10

**Authors:** Eleonora Bargossi, Giulia Tabanelli, Chiara Montanari, Rosalba Lanciotti, Veronica Gatto, Fausto Gardini, Sandra Torriani

**Affiliations:** ^1^Department of Agricultural and Food Sciences, University of BolognaCesena, Italy; ^2^Interdepartmental Center for Industrial Agri-Food Research, University of BolognaCesena, Italy; ^3^Department of Biotechnology, University of VeronaVerona, Italy

**Keywords:** enterococci, tyramine, 2-phenylethylamine, *tyrDC* gene expression, intraspecific variability

## Abstract

The ability to accumulate tyramine and 2-phenylethylamine by two strains of *Enterococcus faecalis* and two strains *Enterococcus faecium* was evaluated in two cultural media added or not with tyrosine. All the enterococcal strains possessed a tyrosine decarboxylase (tyrDC) which determined tyramine accumulation in all the conditions tested, independently on the addition of high concentration of free tyrosine. Enterococci differed in rate and level of biogenic amines accumulation. *E. faecalis* EF37 and *E. faecium* FC12 produced tyramine in high amount since the exponential growth phase, while 2-phenylethylamine was accumulated when tyrosine was depleted. *E. faecium* FC12 and* E. faecalis* ATCC 29212 showed a slower tyraminogenic activity which took place mainly in the stationary phase up to 72 h of incubation. Moreover, *E. faecalis* ATCC 29212 produced 2-phenylethylamine only in the media without tyrosine added. In BHI added or not with tyrosine the *tyrDC* gene expression level differed considerably depending on the strains and the growth phase. In particular, the* tyrDC* gene expression was high during the exponential phase in rich medium for all the strains and subsequently decreased except for *E. faecium* FC12. Even if tyrDC presence is common among enterococci, this study underlines the extremely variable decarboxylating potential of strains belonging to the same species, suggesting strain-dependent implications in food safety.

## Introduction

Tyramine is a biogenic amine (BA) deriving from the microbial decarboxylation of tyrosine. It can have severe acute effects if ingested in excessive amounts with food, causing an hypertensive syndrome known as “cheese reaction,” which consists in peripheral vasoconstriction, increased cardiac output, increased respiration, elevated blood glucose, and release of norepinephrine (McCabe-Sellers et al., 2006; Marcobal et al., 2012).

Lactic acid bacteria (LAB) are among the most efficient producers of tyrosine decarboxylase (tyrDC), the enzyme responsible for tyramine formation. In LAB, BA formation provides metabolic energy and/or acid resistance (Molenaar et al., 1993; Fernández and Zúñiga, 2006). The presence of this enzyme is widespread among all LAB species (Marcobal et al., 2012). However, LAB belonging to the genus *Enterococcus* are recognized as the most efficient tyramine producers (Suzzi and Gardini, 2003; Özogul and Özogul, 2007; Capozzi et al., 2011; Kuley and Özogul, 2011; Ladero et al., 2012).

Tyramine production is considered a species characteristic of *Enterococcus faecalis* and also many strains of *E. faecium* possess this ability (Ladero et al., 2012). The *E. faecalis tyrDC* region was the first tyrDC locus described in prokaryotes (Connil et al., 2002). In *E. faecalis*, upstream the *tyrDC* gene, an ORF can be found (*tyrS*), responsible for a tyrosyl tRNA synthetase involved in an ATP-dependent activation of tyrosine by forming an enzyme-bound tyrosyl-adenylate intermediate (Marcobal et al., 2012). This tyrS could act as a sensor of the intracellular tyrosine pool to regulate tyrosine decarboxylation (Linares et al., 2012). The ORF (*tyrP*) located downstream of *tyrDC* encodes a tyrosine-tyramine antiporter. The three genes are co-transcribed in some strain (Connil et al., 2002; Marcobal et al., 2012). In addition, in enterococci downstream of *tyrP* an ORF was found related to a gene encoding for an Na^+^/H^+^ antiporter (*nhaC-2*) (Pessione et al., 2009; Marcobal et al., 2012). It has been demonstrated that the tyrDC of many tyraminogenic LAB, and especially enterococci, can decarboxylate, although with a lower efficiency, phenylalanine producing 2-phenylethylamine, a BA with characteristics very similar to tyramine (Marcobal et al., 2006).

Enterococci occur in many different habitats and, due to their association with the gastrointestinal tract, they are often contaminant in food of animal origin (Franz et al., 2003, 2011). When present in the raw material, enterococci can survive to the fermentation process and can be found in fermented foods such as sausages and cheeses in which they can have a relevant role during ripening (Giraffa, 2003; Franz et al., 2011). Due to their salt and pH tolerance, as well as their ability to grow over a wide temperature range, these LAB are particularly competitive especially when the environmental conditions become harsher, and can be a relevant component of the ripening microbiota of fermented foods. Their beneficial technological properties and their positive impact on ripening and aroma formation in fermented sausages, cheeses, and olives are reported by several authors. In addition, some strains showed probiotic features, while many enterococci produce bacteriocins able to limit the growth of pathogenic and spoilage microorganisms (Fisher and Phillips, 2009; Franz et al., 2011).

On the other hand, enterococci are among the most common nosocomial pathogens and they can be responsible for endocarditis, bacteremia, as well as urinary tract, central nervous system, intra-abdominal, and pelvic infections. In addition, enterococci are also known for their multiple antibiotic resistance (including vancomycin), which is in some case carried on mobile genetic elements transferable to other microorganisms (Klein, 2003). Moreover, several enterococci virulence factors have been described, such as cytolysins, aggregation substances, and gelatinase extracellular surface proteins (Foulquié Moreno et al., 2006). Finally, the presence of excessive content of tyramine in cheese and fermented meat is often attributed to these microorganisms (Joosten and Northolt, 1989; Suzzi and Gardini, 2003; Foulquié Moreno et al., 2006; Komprda et al., 2008).

The aim of this research was to study the tyramine and 2-phenylethylamine accumulation by four tyraminogenic strains of *Enterococcus*, two belonging to the species *E. faecalis* (EF37 and ATCC 29212) and two to the species *E. faecium* (FC12 and FC643). The ability to accumulate BAs was tested in a rich cultural medium, which does not limit enterococcal growth, and in a poor medium enhancing BA production (Bover-Cid and Holzapfel, 1999). Both media were tested with or without the addition of the precursor (tyrosine). In addition, the *tyrDC* gene expression of the four enterococci was analyzed by reverse transcription-quantitative real time PCR (RT-qPCR) during growth in rich medium in presence or not of the precursor.

## Materials and Methods

### Enterococcal Strains and Growth Conditions

The strains *E. faecalis* EF37 and ATCC 29212, *E. faecium* FC12, and FC643 were used in this work. *E. faecalis* EF37 and *E. faecium* FC12 were previously isolated from traditional Italian cheese while *E. faecium* FC643 were isolated from silage. The strains were stored in 20% (w/v) glycerol at -80°C and pre-cultivated for 24 h at 37°C in BHI Broth (Oxoid, Basingstoke, UK) added with 800 mg/l of tyrosine (Sigma–Aldrich, Gallarate, Italy).

After 24 h of pre-cultivation, the microorganisms were inoculated, at a concentration of approximately 6.5 log CFU/ml, in BHI Broth and in Bover-Cid and Holzapfel broth, a medium proposed to highlight the BA formation (BAM; Bover-Cid and Holzapfel, 1999), added or not with 800 mg/l of tyrosine and incubated at 37°C for 96 h. At defined times (1, 2, 3, 4, 5, 6, 7, 8, 24, 48, 72, and 96 h), the changes of optical density at 600 nm (OD_600_) were monitored. The modification of pH was determined by a pHmeter Basic 20 (Crison Instruments, Barcelona, Spain).

The maximum cell concentration reached in stationary phase was determined after 24 h of incubation by plate counting enterococci onto BHI agar. In addition, 2 ml aliquots of each culture was centrifuged at 3000 rpm for 10 min and the obtained cell pellets were frozen at -80°C.

### Growth Parameters

The evaluation of enterococcal growth in the different media was performed by measuring the OD_600_ with a UV–VIS spectrophotometer (UV-1204, Shimadzu Corporation, Kyoto, Japan) with plastic cuvettes (1.5 ml). The OD_600_ data were fitted with the Gompertz equation as modified by Zwietering et al. (1990).

$$y=k+Ae^{-e\left[\left(\frac{\mu \,\;\max^{e}}{A}\right)(\lambda -t)+1\right]}$$

where *y* is the OD_600_ at time t, *A* represent the maximum OD_600_ value reached, μ_max_ is the maximum OD_600_ increase rate in exponential phase and λ is the lag time.

### Biogenic Amine Determination

The BA were determined after 4, 8, 24, 48, 72, and 96 h of incubation. The cultures were centrifuged at 10000 rpm for 10 min at 10°C, and the supernatants were used for BAs determination by HPLC after derivatization with dansyl-chloride (Sigma–Aldrich, Gallarate, Italy) according to Martuscelli et al. (2000). The BA content was analyzed using a PU-2089 Intelligent HPLC quaternary pump, Intelligent UV–VIS multiwavelength detector UV 2070 Plus (Jasco Corporation, Tokio, Japan) and a manual Rheodyne injector equipped with a 20 μl loop (Rheodyne, Rohnert Park, CA, USA). The quantification was performed according to Tabanelli et al. (2012) and the amount tyramine and 2-phenylethylamine were expressed as mg/ml by reference to a calibration curve obtained with standard solutions.

### Nucleic Acid Extraction from Enterococcal Cultures

Total DNA was isolated from cell pellets by using the Wizard Genomic DNA purification system (Promega Corporation, Madison, WI), following the manufacturer’s instructions.

For total RNA extraction, cells were washed twice with 500 μl of sterile diethyl pyrocarbonate (DEPC)-treated water and shaken three times at the maximum speed for 30 s at 10-s intervals with 500 μl of LETS (200 mM LiCl, 20 mM EDTA, 20 mM Tris, 0,4% SDS, 0,1% DEPC), 500 mg of 450 μm-diameter glass beads (Sigma–Aldrich), 500 μl of phenol pH 4.7-chloroform-isoamyl alcohol (25:24:1 v/v; Sigma–Aldrich) in a cell disrupter (Mini-BeadBeater, BioSpec Products, Bartlesville, OK, USA). After centrifugation (4°C, 13000 rpm, 10 min), the supernatant was twice treated with 600 μl of chloroform-isoamyl alcohol (24:1 v/v; Sigma–Aldrich), added with 60 μl of 3 M sodium acetate, 1 ml of ice-cold absolute ethanol and left for 1 h at -80°C. Total RNA was pelleted by centrifugation at 13000 rpm for 5 min at 4°C, washed with 200 μl of ethanol 70%, and dissolved in 30 μl of sterile water (RNAse- and DNAse-free).

DNA elimination was performed using 50 U of RNase-free DNase I recombinant (Roche Diagnostic, Germany) in 50 μl of DNAse reaction buffer (40 mM Tris-HCl, 10 mM NaCl, 6 mM MgCl_2_, 1 mM CaCl_2_, pH 7.9) for 30 min at 25°C. A PCR assay was carried out to check for any contaminating DNA, and, when necessary, the DNase treatment was repeated.

DNA and RNA integrity, concentration, and purity were checked by electrophoresis on a 1,5% (wt/vol) agarose gel and by measurement with the NanoDropTM Lite Spectrophotometer (Thermo Fisher Scientific Inc., Waltham, MA, USA).

DNA and DNA-free RNA samples were stored at -20°C and -80°C, respectively, until use.

### PCR Amplification and Expression of the *tyrDC* Gene

A tyrDC fragment of about 336 bp was amplified using the primers DEC5 (5^′^-CGT TGT TGG TGT TGT TGG CAC NAC NGA RGA RG-3^′^) and DEC3 (5^′^-CCG CCA GCA GAA TAT GGA AYR TAN CCC AT-3^′^), following the PCR conditions described previously (Torriani et al., 2008). PCR product was visualized on a 2% agarose gel.

Total cDNA was synthesized from 1 μg of RNA using the ImProm-IITM Reverse Transcriptase kit (Promega, USA), following the manufacturer’s recommendations.

The expression level of the tyrDC gene was analyzed by a RT-qPCR assay with primers TYR3f (5^′^-CGT ACA CAT TCA GTT GCA TGG CAT-3^′^) and TYR4r (5^′^-ATG TCC TAC TTC TTC TTC CAT TTG-3^′^); thermo cycler, reaction mixture, and amplification program were described in Torriani et al. (2008), as well as the procedure of the absolute quantification of the tyrDC copies number.

### Statistical Analysis

The growth model was fitted using the statistical package Statistica for Windows 6.1 (Statsoft Italia, Vigonza, Italy).

## Results

### Growth and pH Modification in Cultural Media

The growth of four enterococcal strains, i.e., *E. faecalis* EF37 and ATCC 29212, and *E. faecium* FC12 and FC643, was monitored by measuring the OD_600_ increase in the absence or in the presence of tyrosine (800 mg/l) added in BHI and BAM media. The OD_600_ changes were modeled with the Gompertz equation (Zwietering et al., 1990) and the estimates of the parameters are reported in **Table 1**.

**Table 1:** Gompertz equation parameters for enterococcal growth measured as OD_600_.

Strain	Cultural medium	Gompertz equation parameters^a^			*R*^2^	Residual mean square error (RMSE)	Maximum cell concentration (log CFU/ml)
		*A*	*µ*_max_	λ			
EF37	BHI + tyr^b^	0.947	0.767	2.532	0.977	0.042	9.40 (±0.13)
	BHI	1.029	0.601	2.030	0.954	0.068	9.42 (±0.13)
	BAM + tyr^c^	0.600	0.171	2.619	0.991	0.024	8.95 (±0.14)
	BAM	0.803	0.192	2.121	0.993	0.031	9.04 (±0.11)
ATCC 29212	BHI + tyr	0.899	0.494	2.399	0.989	0.013	9.30 (±0.15)
	BHI	1.014	0.632	2.109	0.990	0.069	9.48 (±0.19)
	BAM + tyr	0.544	0.191	3.455	0.989	0.049	8.52 (±0.15)
	BAM	0.788	0.252	2.544	0.992	0.043	8.80 (±0.14)
FC12	BHI + tyr	1.119	1.170	3.589	0.996	0.029	9.34 (±0.12)
	BHI	1.095	0.559	2.848	0.995	0.054	9.35 (±0.16)
	BAM + tyr	0.362	0.132	1.882	0.989	0.037	8.33 (±0.14)
	BAM	0.425	0.124	2.077	0.993	0.039	8.31 (±0.17)
FC643	BHI + tyr	1.114	0.566	2.876	0.983	0.047	9.38 (±0.09)
	BHI	1.191	0.584	2.158	0.989	0.036	9.52 (±0.13)
	BAM + tyr	0.739	0.177	1.635	0.992	0.028	8.46 (±0.20)
	BAM	0.807	0.187	1.445	0.993	0.028	8.72 (±0.11)

*R*^2^ and RMSE are given as diagnostics of the regression. The maximum cell concentrations (expressed as log CFU/ml) at the beginning of the stationary phase is also reported.

^a^*A*, maximum OD_600_ value reached; µ_max_, maximum OD_600_ increase rate in exponential phase (OD_600_/h); λ, lag phase duration (h).

^b^BHI Broth plus 800 mg/l tyrosine.

^c^Bover-Cid and Holzapfel medium (BAM) plus 800 mg/l tyrosine.

When inoculated in BHI medium, all the strains reached the maximum value of OD_600_ after 8 h of incubation at 37°C, independently on the presence of tyrosine. Given the high initial inoculums (about 6 log CFU/ml), the lag phase (λ) was always very short and it was followed by a sharp increase of OD_600_, whose maximum values (estimated by the *A* parameter of the equation) ranged between 0.9 and 1.2. In particular, the absence of tyrosine favored the reaching of higher OD_600_ values for the two *E. faecalis* strains, and for *E. faecium* FC643. By contrast, no differences in the maximum OD_600_ were found for *E. faecium* FC12 in relation to the presence of tyrosine added.

**Table 1** reports also the cell counts detected in the stationary phase (determined after 24 h incubation). In BHI no differences were detected in relation to the strain and to the addition of tyrosine. In fact, the counts revealed final cell concentrations comprised between 9.30 and 9.52 log CFU/ml.

All the strains, as expected, showed lower growth extent in BAM if compared with BHI. BAM is considered a poor medium and the energetic supply provided by aminoacid decarboxylation (and, consequently, by BA formation) became fundamental to support microbial growth. The more stringent conditions provided by this medium are reflected also in the lower OD_600_ reached in the stationary phase. Anyway, the OD_600_ was always higher in the BAM not supplemented with tyrosine (**Table 1**). Also cell counts in stationary phase confirmed this behavior and were always higher in the medium without tyrosine, with the exception of the strain* E. faecium* FC643 in which no differences related to the presence of the aminoacid were found.

**FIGURE 1:**
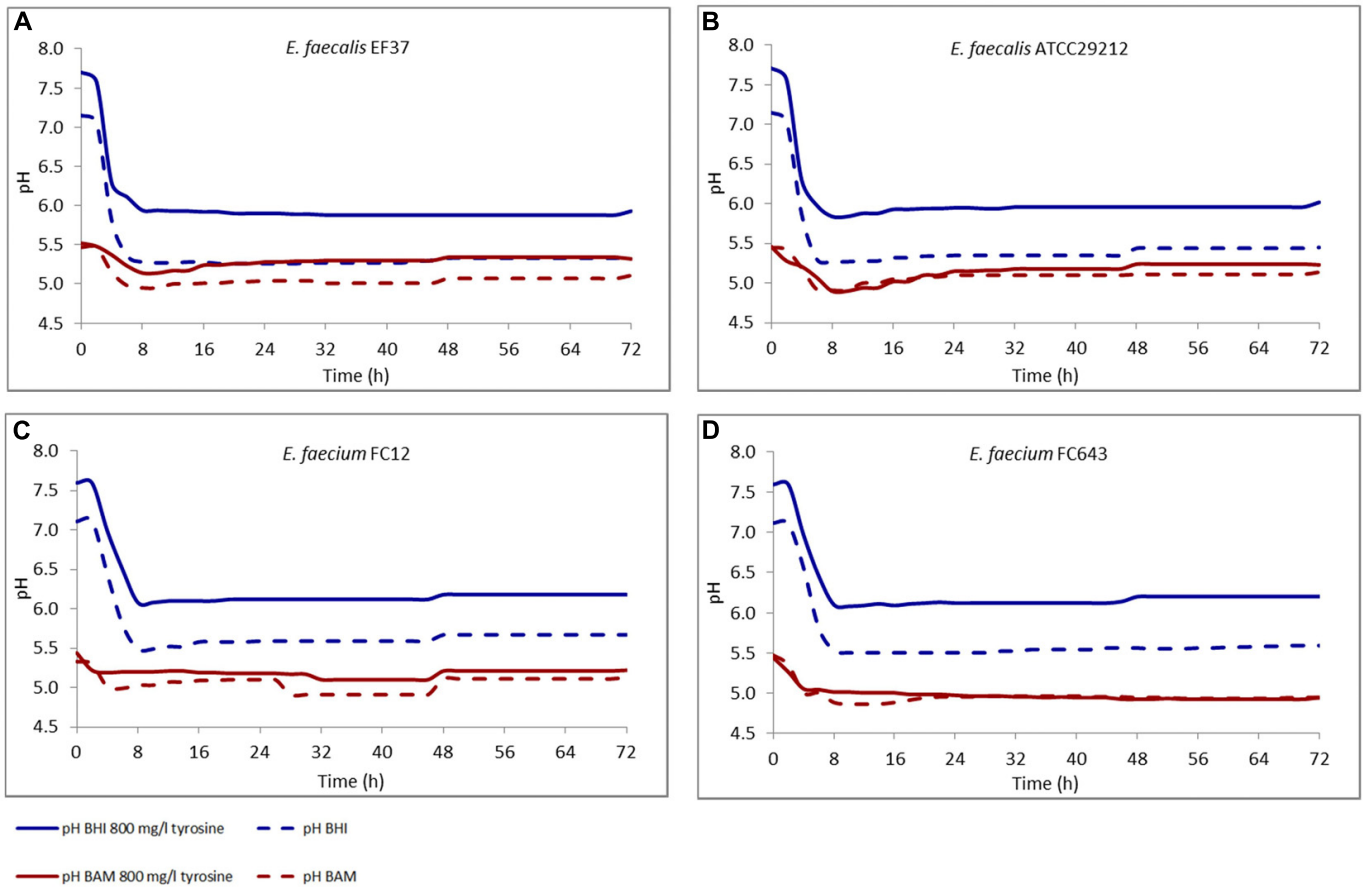
**Modification of the pH in BHI and Bover-Cid medium (BAM) with or without tyrosine. (A)** *E. faecalis* EF37, **(B)** *E. faecalis* ATCC 29212, **(C)** *E. faecium* FC12, **(D)** *E. faecium* FC643.

During incubation, the pH of the media was also monitored and the data are reported in **Figure 1**. The results in BHI were specular to the growth curves for all the strains, and the pH decrease within the first 8 h was of about 1.5 units, and was quite constant during the remaining incubation period. In the samples added with tyrosine the pH value was higher of about 0.5 units, both at the beginning and at the end of incubation.

As far as BAM, the initial pH was 5.5 and only a slight decrease was observed in the first 8 h of incubation. This behavior can be attributed to the higher buffering potential of the medium. After the first 8 h, the pH showed a slight increase determined by the accumulation of BAs.

### Biogenic Amine Production in the Cultural Media Not Added with Tyrosine

**FIGURE 2:**
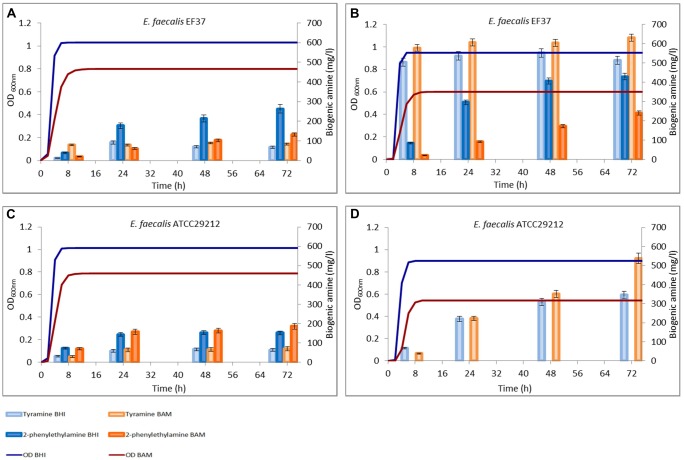
**Amounts of tyramine and 2-phenylethylamine produced during the growth in BHI and Bover-Cid medium (BAM) without **(A,C)** and with **(B,D)** tyrosine addition. (A,B)** *Enterococcus faecalis* EF37, **(C,D)** *E. faecalis* ATCC 29212.

**FIGURE 3:**
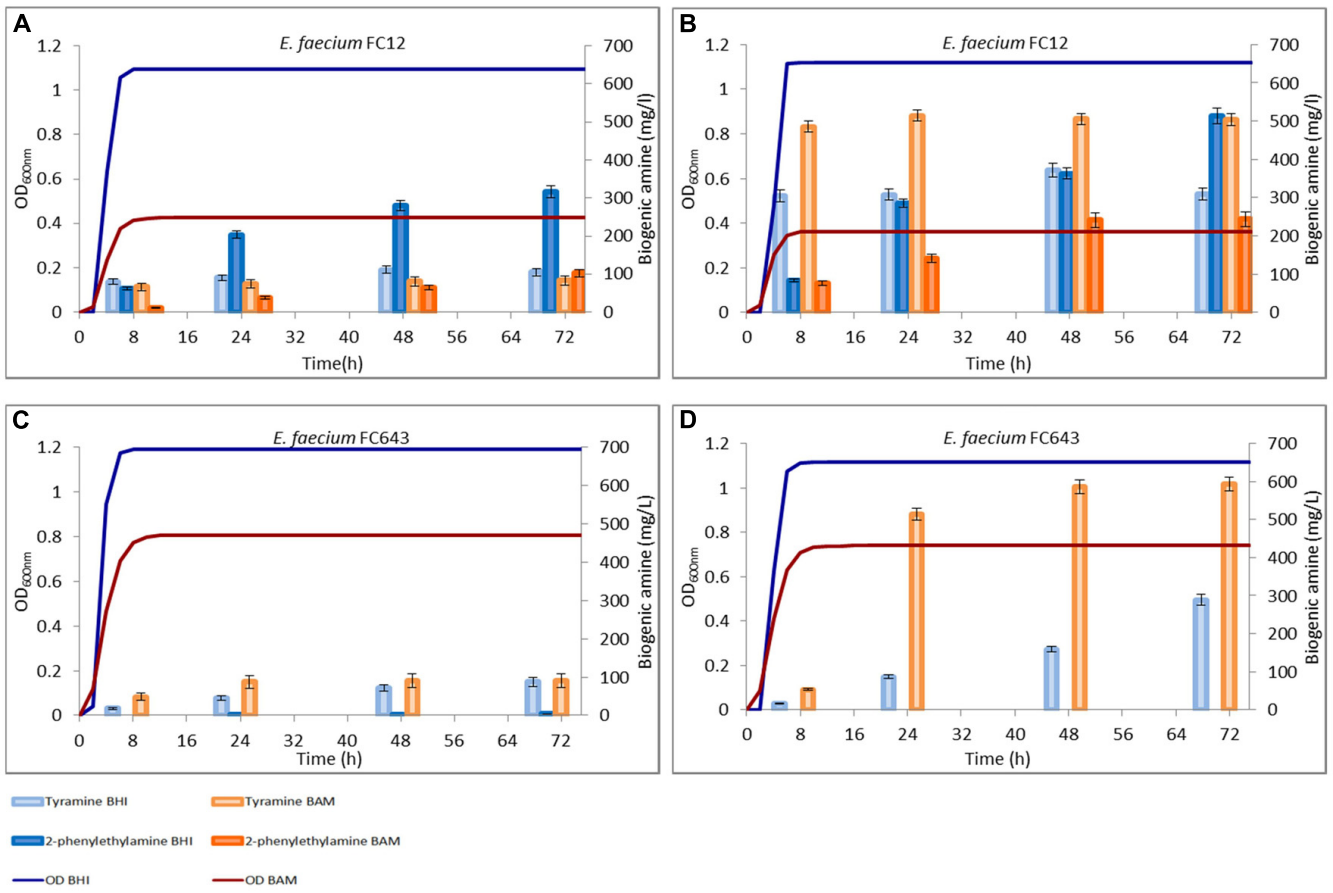
**Amounts of tyramine and 2-phenylethylamine produced during the growth in BHI and Bover-Cid medium (BAM) without **(A,C)** and with **(B,D)** tyrosine addition. (A,B)** *Enterococcus faecium* FC12, **(C,D)** *E. faecium* FC643.

Both the media used for the trials contained, in different amount, aminoacid sources (proteins and peptides) among which precursors for tyrDC were present, allowing a decarboxylase activity of the strains also in the absence of tyrosine added. The amounts of tyramine and 2-phenylethylamine produced during the growth in these media are reported in **Figures 2** and **3**.

*Enterococcus faecalis* EF37 was able to produce tyramine in both media. However, the final amount after 72 h did not exceed 70 mg/l in BHI and 90 mg/l in BAM (**Figure 2A**). These values did not significantly change prolonging the incubation up to 96 h (data not shown). The maximum tyramine concentration was observed in the samples taken after 24 h of incubation. Also 2-phenylethylamine was produced under the same conditions and this BA was gradually accumulated reaching a concentration of about 270 mg/l in BHI and 130 mg/l in BAM after 72 h. *E. faecalis* ATCC 29212 showed an analogous decarboxylating activity even if lower tyramine amounts were produced in both media (**Figure 2C**).

A similar trend was observed for *E. faecium* FC12, though this strain accumulated in BHI higher concentrations of both the BAs while an opposite trend was observed for BAM (**Figure 3A**). Finally, *E. faecalis* FC643 showed the minor BA production in both the media and produced only traces of 2-phenylethylamine only in BHI (**Figure 3C**).

### Biogenic Amine Production in the Cultural Media Added with Tyrosine

**Figures 2** and **3** report also the tyramine and 2-phenylethylamine accumulation when tyrosine (800 mg/l) was added to the two media. *E. faecalis* EF37 and *E. faecium* FC12 accumulated the maximum tyramine concentration within the first 8 h of incubation both in BHI and BAM and the reaching of the stationary phase did not further increase significantly these amounts (**Figures 2B** and **3B**, respectively).

*Enterococcus faecalis* EF37 was the most efficient strain in the conversion of tyrosine to tyramine and the final concentration of this BA was about 515 mg/l in BHI and 620 mg/l in BAM. The behavior of *E. faecium* FC12 was similar, but the final amounts of tyramine were lower than *E. faecalis* EF37, i.e., 505 and 360 mg/l of BA were produced in BAM and BHI, respectively. In addition, both the strains began to produce 2-phenylethylamine only after 8 h of incubation, i.e., when stationary phase and the maximum amount of tyramine have been reached. The 2-phenylethylamine accumulation increased during subsequent incubation and reached its maximum level at 72 h. About 513 and 428 mg/l of this amine were produced in BHI by *E. faecium* FC12 and *E. faecalis* EF37, respectively, while lower concentrations were detected in BAM (about 230 mg/l for both the strains). In any case, these amounts were higher if compared with the 2-phenylethylamine produced in the same media in the absence of the tyrosine addition.

The presence of tyrosine determined a different behavior in the sample inoculated with *E. faecalis* ATCC 29212. Indeed, the growth of this strain was characterized by a slowed rate of accumulation of tyramine. This BA was mainly produced when the cells reached the stationary phase and amounts of about 325 and 510 mg/l were detected only after 72 h of incubation in BHI and BAM, respectively. In addition, no 2-phenylethylamine was produced in both the conditions, even if this ability was displayed in the media without the addition of precursor (**Figure 2D**).

*Enterococcus faecium* FC643 showed a similar behavior and reached in BAM a double concentration of tyramine with respect to BHI (592 mg/l vs. 288 mg/l). Also *E. faecium* FC643 did not accumulate 2-phenylethylamine when tyrosine was added to the media (**Figure 3D**).

### Expression of the *tyrDC* Gene in BHI Added or Not with Tyrosine

All the enterococcal strains produced a 336-bp fragment characteristic of the *tyrDC* gene with the degenerate primers DEC5/DEC3 (data not shown), in accordance with their ability to accumulate tyramine. Therefore, the expression of the *tyrDC* gene could be evaluated by RT-qPCR during a period of 72 h. The expression of *tyrDC* gene has been evaluated during the enterococcal growth in BHI with or without tyrosine added.

As shown in **Table 2**, the *tyrDC* gene expression level differed considerably depending on the the strains and the growth phase. In general, the two strains with the most effective tyraminogenic activity showed since the beginning of incubation a level of transcript considerably higher than the other strains. It is important to note that the amount of *tyrDC* transcription is given as absolute value and the higher amount detected after 2 h did not correspond with the reaching of the maximum cell counts during incubation. Thus, the cell showed a considerably higher transcription activity during their early exponential phase.

**Table 2:** Tyrosine decarboxylase (*tyrDC)* gene expression level for enterococcal strains grown in BHI Broth and BHI Broth added with 800 mg/l tyrosine during 72 h.

Time (h)	Log (copies/µg cDNA)							
	*Enterococcus faecalis* EF37		*E. faecalis* ATCC 29212		*E. faecium* FC12		*E. faecium* FC643	
	BHI	BHI + tyr	BHI	BHI + tyr	BHI	BHI + tyr	BHI	BHI + tyr
2	5.08 (±0.02)	4.79 (±0.06)	3.11 (±0.07)	2.60 (±0.08)	4.98 (±0.22)	3.91 (±0.18)	3.86 (±0.02)	2.79 (±0.02)
4	4.87 (±0.01)	6.11 (±0.02)	3.06 (±0.14)	3.25 (±0.02)	3.45 (±0.01)	3.49 (±0.07)	2.19 (±0.11)	2.65 (±0.01)
8	5.22 (±0.05)	5.03 (±0.05)	2.23 (±0.09)	2.07 (±0.09)	3.37 (±0.42)	2.98 (±0.19)	1.98 (±0.25)	0.98 (±0.34)
24	2.42 (±0.07)	4.15 (±0.05)	1.10 (±0.45)	1.61 (±0.17)	3.42 (±0.07)	4.61 (±0.24)	1.65 (±0.29)	0.84 (±0.06)
48	2.81 (±0.03)	3.38 (±0.03)	1.02 (±0.04)	1.83 (±0.01)	3.08 (±0.63)	4.37 (±0.02)	2.04 (±0.02)	1.24 (±0.02)
72	1.01 (±0.29)	4.10 (±0.12)	1.01 (±0.29)	1.61 (±0.02)	3.52 (±0.27)	3.47 (±0.68)	1.63 (±0.02)	1.11 (±0.20)

*Enterococcus faecalis* EF37 displayed the highest level of *tyrDC* transcription [up to 5–6 log (copies/μg cDNA)] during the exponential phase of growth (2–5 h) in both media, according with its great ability to accumulate tyramine. After that, a significant decrease was found in BHI without tyrosine, while the *tyrDC* mRNA was rather high in BHI added with tyrosine, thus supporting the sequential 2-phenylethylamine production.

The *tyrDC* gene expression of *E. faecalis* ATCC 29212 was always lower (about two log units in the first 8 h) if compared with *E. faecalis* EF37. Its expression was higher in BHI without added tyrosine at the beginning of the growth and transcription decreased drastically after 8 h.

The strain *E. faecium* FC12, although exhibiting a phenotypic behavior analogous to *E. faecalis* EF37, showed a very different trend of the *tyrDC* gene expression. Indeed, the transcript level in both media was not related to the phase of growth, but it was rather constant during the entire incubation period. For this strain the high level of transcript after 24 h could determine the increase of 2-phenylethyamine in both the media.

Finally, *E. faecium* FC643 exhibited a *tyrDC* gene expression behavior similar to *E. faecalis* ATCC 29212. However, it was characterized by the lowest levels of *tyrDC* transcription after 4 h of incubation.

## Discussion

All the enterococcal strains used in these trials possessed an active tyrDC which determined tyramine accumulation (even if at different level) in all the conditions tested, independently on the addition of high concentration of free tyrosine. This fact clearly indicated the possibility of the enterococci to decarboxylate amino acids present in the proteic and peptidic ingredients used for media preparation.

Recently, Liu et al. (2014a) showed that the activity of purified recombinant tyrDCs of the strains *E. faecalis* R612Z1 and *E. faecium* R615Z1 was similar, and that the enzymes exhibited higher specificity for tyrosine than for phenylalanine. However, in our trials, different behaviors of tyramine and 2-phenylethylamine accumulation were observed in relation to the strain. In particular, strains belonging to the same species (*E. faecalis* or *E. faecium*) were characterized by different responses indicating that, if the presence of tyrDC is common among enterococci, the decarboxylating potential can be extremely variable. The transcriptional analyses of the gene* tyrDC* confirmed these observations. In recent years, a number of studies have been conducted to evaluate the gene expression level of amino acid decarboxylases on the BAs accumulation in different food and model systems (Gardini et al., 2008; Arena et al., 2011; La Gioia et al., 2011; Rossi et al., 2011; Liu et al., 2014b). However, up to now no studies have compared the *tyrDC* transcript levels in enterococcal strains of the same species. In the present study a variability of the *tyrDC* gene expression in different strains of* E. faecalis* and *E. faecium* was evidenced for the first time. The two strains with the higher decarboxylating potential (*E. faecalis* EF 37 and *E. faecium* FC12) were characterized by a higher transcription of *tyrDC* after 2 h of incubation and the maintenance of a remarkably higher transcription level throughout all the incubation period considered here.

In contrast with Arena et al. (2011), who observed an increase of *tyrDC* transcription in the presence of tyrosine for *Lactobacillus brevis* IOEB 9809 in wine, in our conditions the presence of the precursor in high amounts (800 mg/l) did not enhance the transcript at the beginning of growth, but stimulated a higher transcription during the successive incubation.

Linares et al. (2009) evidenced that the maximization of the transcription of *tyrDC* and *tyrP* in* E. durans* was caused by a tyrosine concentration comprised between 2 and 5 mM. In this framework, the presence of tyramine added (800 mg/kg, i.e., 4.42 mM) should be sufficient to reach a high level of *tyrDC* transcript. Nevertheless, similar and often higher transcripts were obtained also in the medium without the addition of the precursor.

*Enterococcus faecalis* EF37 showed the higher tyramine production and *tyrDC* gene expression in the presence of tyrosine added to the media. In addition, independently on the media, *E. faecalis* EF37 and *E. faecium* FC12 produced also high amount of 2-phenylethylamine, which were significantly higher in the presence of tyrosine added. On the other hand, *E. faecalis* ATCC 29212 and *E. faecium* FC643 were not able to accumulate significantly 2-phenylethylamine when tyrosine was added; however, the same strains produced this BA in reduced amounts in the absence of tyrosine in BHI if compared with the other two strains and in the same magnitude in the poor medium (BAM). The absence of 2-phenylethylamine in BAM and BHI added with tyrosine and inoculated with *E. faecalis* ATCC 29212 and *E. faecium* FC643 reflected the lower efficiency of their tyrDC and could indicate that for these strains the increasing amount of tyramine can lower or inhibit further decarboxylase activities. Concerning transcriptional analysis, the maintenance of the *tyrDC* gene expression in the stationary phase of growth could contribute to enhance 2-phenylethylamine biosynthesis in enterococcal strains when the preferred precursor was depleted.

However, in the case of the strains *E. faecalis* ATCC 29212 and *E. faecium* FC643, that accumulated gradually tyramine, the *tyrDC* gene expression was still present at the end of the incubation period, but 2-phenylethylamine was not produced because tyrosine was not entirely consumed after 72 h growth.

The early production of tyramine by *E. faecalis* EF37 and *E. faecium* FC12 confirms the results of Pessione et al. (2009), who demonstrated that tyrDC activity in *E. faecalis* DISAV1022 reached its maximum level during the exponential growth phase, suggesting that tyrosine decarboxylation was not simply a response to starvation or nutrient depletion typical of the stationary phase.

By contrast, the strains *E. faecalis* ATCC 29212 and *E. faecium* FC643 accumulate great part of tyramine after they reached the stationary phase, independently on the addition of precursor. However, these strains, although their transcript levels were much lower respect to *E. faecalis* EF37, showed a *tyrDC* transcription trend similar to *E. faecalis* EF37. In particular, these profiles, were characterized by an higher expression during the exponential phase followed by a decrease after 8 h of incubation.

Many authors reported the widespread ability of enterococci to produce both tyramine and 2-phenylethylamine (Beutling and Walter, 2002; Aymerich et al., 2006; Bonetta et al., 2008). This characteristic was found also in some lactobacilli (Landete et al., 2007), even if in other case a highly tyrosine selective tyrDC was described (Moreno-Arribas and Lonvaud-Funel, 2001). Marcobal et al. (2006) proved that *tyrDC* gene in *E. faecium* encoded for a functional and dual decarboxylase resulting in tyrosine and phenylalanine decarboxylation. Also Landete et al. (2007) demonstrated that tyrDC present in LAB allowed the production of 2-phenylethylamine. Pessione et al. (2009) carried out a comparative proteomic investigation on *E. faecalis*, which demonstrated a membrane bound tyrDC highly overexpressed during the production of both tyramine and 2-phenylethylamine. According to these authors, a yield of 100% was observed for the conversion of tyrosine, which takes place since the exponential phase. On the other hand, the yield for 2-phenylethylamine was lower (about 10%) and its production occurred in the stationary phase when tyrosine was exhausted. Also other authors observed that phenylalanine is decarboxylated with a reduced efficiency and only when the tyrosine become a limiting substrate (Joosten, 1988; Latorre-Moratalla et al., 2014). Regarding the different amount of tyramine accumulated under the same conditions, it has been demonstrated that the presence of high amounts of tyrosine in the medium can reduce the tyramine production by some LAB (Fernández et al., 2007). In other words, the increase in the availability of the precursor did not necessarily coincide with an increase in the decarboxylation.

The maximum yield for tyrosine conversion is 75.7%; then, in this study a stoichiometric conversion of the aminoacid added should produce a maximum theoretical level of 609 g/l of tyramine. Taking into account that BHI without precursor supports the production of 70–90 mg/l of tyramine, the maximum accumulation of this BA showed by *E. faecalis* EF37 in the presence of tyrosine is lower than this theoretical limit (about 150 mg/l below). However, in these conditions a higher production of 2-phenylethylamine (150 mg/l above the yield in not supplemented BHI) occurred. The overproduction of this BA was observed also in *E. faecium* FC12; however, in this case the results were reached in the presence of lower tyramine concentration. The same trend was also observed in BAM, in which *E. faecalis* EF37 seems to operate an almost complete conversion of the aromatic aminoacids added or naturally present in the medium.

## Conclusion

The presence of *Enterococcus* strains that can decarboxylate tyrosine and 2-phenylalanine is a serious concern in fermented food for consumer’s health. Indeed, even if these activities are common among enterococci, this study underlines the extremely variable decarboxylating potential of strains belonging to the same species, suggesting strain-dependent implications in food safety. In spite of the fact that all the strains tested here had the *tyrDC* gene, the amounts of tyramine (and 2-phenylethylamine) produced was strictly dependent on the amount of its transcription, which was extremely different among the strains. The composition of the media also affected and modulated the amount and ratio of these BAs by tyraminogenic strains, indicating the need of preventive measures to control BAs accumulation in foods. Future researches will be planned to a deep knowledge on the conditions which can favor the production of BAs by enterococci and on the reasons which determine the important differences among the transcripts of the same gene. This could be ascribed to different activity and specificity of tyrDC enzyme or to different regulation mechanisms. In this regards, further studies have to be performed to better explain the genetic and functional basis, and the environmental factors affecting the different decarboxylating potential of the strains.

## Conflict of Interest Statement

The authors declare that the research was conducted in the absence of any commercial or financial relationships that could be construed as a potential conflict of interest.

## References

[B1] Arena M. P. Romano A. Capozzi V. Beneduce L. Ghariani M. Grieco F. (2011). Expression of *Lactobacillus brevis* IOEB 9809 tyrosine decarboxylase and agmatine deiminase genes in wine correlates with substrate availability. *Lett. Appl. Microbiol.* 53 395–402 10.1111/j.1472-765X.2011.03120.x 21740449

[B2] Aymerich T. Martín B. Garriga M. Vidal-Carou M. C. Bover-Cid S. Hugas M. (2006). Safety properties and molecular strain typing of lactic acid bacteria from slightly fermented sausages. *J. Appl. Microbiol.* 100 40–49 10.1111/j.1365-2672.2005.02772.x 16405683

[B3] Beutling D. M. Walter D. (2002). 2-phenylethylamine formation by enterococci in vitro. *Eur. Food Res. Technol.* 215 240–242 10.1007/s00217-002-0525-y

[B4] Bonetta S. Bonetta S. Carraro E. Coïsson J. D. Travaglia F. Arlorio M. (2008). Detection of biogenic amine producer bacteria in a typical italian goat cheese. *J. Food Protect.* 1 205–209. 10.4315/0362-028x-71.1.205 18236686

[B5] Bover-Cid S. Holzapfel W. H. (1999). Improved screening procedure for biogenic amine production by lactic acid bacteria. *Int. J. Food Microbiol.* 53 33–41 10.1016/S0168-1605(99)00152-X 10598112

[B6] Capozzi V. Ladero V. Beneduce L. Fernández M. Alvarez M. A. Bach B. (2011). Isolation and characterization of tyramine-producing *Enterococcus faecium* strain from red wine. *Food Microbiol.* 28 434–439 10.1111/j.1472-765X.2011.03120.x 21356448

[B7] Connil N. Le Breton Y. Dousset X. Auffray Y. Rincé A. Prévost H. (2002). Identification of the *Enterococcus faecalis* tyrosine decarboxylase operon involved in tyramine production. *Appl. Environ. Microb.* 68 3537–3544 10.1128/AEM.68.7.3537-3544.1002 PMC126796 12089039

[B8] Fernández M. Linares D. M. Rodríguez A. Alvarez M. A. (2007). Factors affecting tyramine production in *Enterococcus durans* IPLA 655. *Appl. Microbiol. Biotechnol.* 73 1400–1406 10.1007/s00253-006-0596-y 17043827

[B9] Fernández M. Zúñiga M. (2006). Amino acid catabolic pathways of lactic acid bacteria. *Crit. Rev. Microbiol.* 32 155–183 10.1080/10408410600880643 16893752

[B10] Fisher K. Phillips C. (2009). The ecology, epidemiology and virulence of *Enterococcus*. *Microbiology* 155 1749–1757 10.1099/mic.0.026385-0 19383684

[B11] Foulquié Moreno M. R. Sarantinopoulos P. Tsakalidou E. De Vuyst L. (2006). The role and application of enterococci in food and health. *Int. J. Food Microbiol.* 106 1–24 10.1016/j.ijfoodmicro.2005.06.026 16216368

[B12] Franz C. M. Huch M. Abriouel H. Holzapfel W. Gálvez A. (2011). Enterococci as probiotics and their implications in food safety. *Int. J. Food Microbiol.* 151 125–140 10.1016/j.ijfoodmicro.2011.08.014 21962867

[B13] Franz C. M. Stiles M. E. Schleifer H. K. Holzapfel W. (2003). Enterococci in food-a conundrum for food safety. *Int. J. Food Microbiol.* 88 105–122 10.1016/S0168-1605(03)00174-0 14596984

[B14] Gardini F. Bover-Cid S. Tofalo R. Belletti N. Gatto V. Suzzi G. (2008). Modeling the aminogenic potential of *Enterococcus faecalis* EF37 in dry fermented sausages through chemical and molecular approaches. *Appl. Environ. Microb.* 74 2740–2750 10.1128/AEM.02267-07 PMC2394872 18296537

[B15] Giraffa G. (2003). Functionality of enterococci in dairy products. *Int. J. Food Microbiol.* 88 215–222 10.1016/S0168-1605(03)00183-1 14596993

[B16] Joosten H. M. L. J. (1988). Conditions allowing the formation of biogenic amine in cheese: 4. Factors influencing the amounts formed. *Neth. Milk Dairy J.* 41 329–357.

[B17] Joosten H. M. L. J. Northolt M. D. (1989). Detection, growth and amine-producing capacity of lactobacilli in cheese. *Appl. Environ. Microb.* 55 2356–2359. 10.1128/aem.55.9.2356-2359.1989 PMC203080 16348016

[B18] Klein G. (2003). Taxonomy, ecology and antibiotic resistance of enterococci from food and the gastro-intestinal tract. *Int. J. Food Microbiol.* 88 123–131 10.1016/S0168-1605(03)00175-2 14596985

[B19] Komprda T. Burdychová R. Dohnal V. Cwiková O. Sládková P. (2008). Some factors influencing biogenic amines and polyamines content in Dutch-type semi-hard cheese. *Eur. Food Res. Technol.* 227 29–36 10.1007/s00217-007-0688-7

[B20] Kuley E. Özogul F. (2011). Synergistic and antagonistic effect of lactic acid bacteria on tyramine production by food-borne pathogenic bacteria in tyrosine decarboxylase broth. *Food Chem.* 127 1163–1168 10.1016/j.foodchem.2011.07.118 25214109

[B21] La Gioia F. Rizzotti L. Rossi F. Gardini F. Tabanelli G. Torriani S. (2011). Identification of a tyrosine decarboxylase gene (tdcA) in *Streptococcus thermophilus* 1TT45 and analysis of its expression and tyramine production in milk. *Appl. Environ. Microb.* 77 1140–1144 10.1128/AEM.01928-10 PMC3028712 21131517

[B22] Ladero V. Fernández M. Calles-Enríquez M. Sánchez-Llana E. Cañedo E. Martin M. C. (2012). Is the production of the biogenic amines tyramine and putrescine a species-level trait in enterococci? *Food Microbiol*. 30 132–138 10.1016/j.fm.2011.12.016 22265293

[B23] Landete J. M. Pardo I. Ferrer S. (2007). Tyramine and phenylethylamine production among lactic acid bacteria isolated from wine. *Int. J. Food Microbiol.* 115 364–368 10.1016/j.ijfoodmicro.2006.10.051 17307265

[B24] Latorre-Moratalla M. L. Bover-Cid S. Bosch-Fusté J. Veciana-Nogués M. T. Vidal-Carou C. (2014). Amino acid availability as an influential factor on the biogenic amine formation in dry fermented sausages. *Food Control.* 36 76–81 10.116/j.foodcont.2013.07.038

[B25] Linares D. M. Fernández M. Del-Rio B. Ladero V. Martin M. C. Alvarez M. A. (2012). The tyrosyl-tRNA synthetase like gene located in the tyramine biosynthesis cluster of *Enterococcus durans* is transcriptionally regulated by tyrosine concentration and extracellular pH. *BMC Microbiol.* 12:23 10.1186/1471-2180-12-23 PMC3315439 22333391

[B26] Linares D. M. Fernández M. Martín M. C. Alvarez M. A. (2009). Tyramine biosynthesis in *Enterococcus durans* is trascriptionally regulated by the extracellular pH and tyrosine concentration. *Microb. Biotechnol.* 2 625–633 10.1111/j.1751-7915.2009.00117.x 21255297 PMC3815318

[B27] Liu F. Xu W. Du L. Wang D. Zhu Y. Geng Z. (2014a). Heterologous expression and characterization of tyrosine decarboxylase from *Enterococcus faecalis* R612Z1 and *Enterococcus faecium* R615Z1. *J. Food Protect.* 77 592–598 10.4315/0362-028X.JFP13-326 24680070

[B28] Liu F. Du L. Wu H. Wang D. Zhu Y. Geng Z. (2014b). Effects of storage temperature on tyramine production by *Enterococcus faecalis* R612Z1 in water-boiled salted ducks. *J. Food Protect.* 77 1804–1808 10.4315/0362-028X.JFP-14-141 25285502

[B29] Marcobal A. De Las Rivas B. Landete J. M. Tabera L. Muñoz R. (2012). Tyramine and phenylethylamine biosynthesis by food bacteria. *Crit. Rev. Food Sci.* 52 448–467 10.1080/10408398.2010.500545 22369263

[B30] Marcobal A. de las Rivas B. Muñoz R. (2006). First genetic characterization of a bacterial β-phenylethylamine biosynthetic enzyme in *Enterococcus faecium* RM58. *FEMS Microbiol. Lett.* 258 144–149 10.1111/j.1574-6968.2006.00206.x 16630269

[B31] Martuscelli M. Crudele M. A. Gardini F. Suzzi G. (2000). Biogenic amine formation and oxidation by *Staphylococcus xylosus* strains from artisanal fermented sausages. *Lett. Appl. Microbiol.* 31 228–232 10.1046/j.1365-2672.2000.00796.x 10972734

[B32] McCabe-Sellers B. Staggs C. G. Bogle M. L. (2006). Tyramine in foods and monoamine oxidase inhibitor drugs: a crossroad where medicine, nutrition, pharmacy, and food industry converge. *J. Food Comp. Anal.* 19 S58–S65 10.1016/j.jfca.2005.12.008

[B33] Molenaar D. Bosscher J. S. ten Brink B. Driessen A. J. Konings W. N. (1993). Generation of a proton motive force by histidine decarboxylation and electrogenic histidine/histamine antiport in *Lactobacillus buchneri*. *J. Bacteriol.* 175 2864–2870. 8387991 10.1128/jb.175.10.2864-2870.1993 PMC204603

[B34] Moreno-Arribas V. Lonvaud-Funel A. (2001). Purification and characterization of tyrosine decarboxylase of *Lactobacillus brevis* IOEB 9809 isolated from wine. *FEMS Microbiol. Lett.* 195 103–107 10.1111/j.1574-6968.2001.tb10505.x 11167003

[B35] Özogul F. Özogul Y. (2007). The ability of biogenic amines and ammonia production by single bacterial cultures. *Eur. Food Res. Technol.* 225 385–394 10.1007/s00217-006-0429-3

[B36] Pessione E. Pessione A. Lamberti C. Coïsson D. J. Riedel K. Mazzoli R. (2009). First evidence of a membrane-bound, tyramine and β-phenylethylamine producing, tyrosine decarboxylase in *Enterococcus faecalis*: a two-dimensional electrophoresis proteomic study. *Proteomics* 9 2695–2710 10.1002/pmic.200800780 19405032

[B37] Rossi F. Gardini F. Rizzotti L. La Gioia F. Tabanelli G. Torriani S. (2011). Quantitative analysis of histidine decarboxylase gene (hdcA) transcription and histamine production by *Streptococcus thermophilus* PRI60 under conditions relevant to cheese making. *Appl. Environ. Microb.* 77 2817–2822 10.1128/AEM.02531-10 PMC3126354 21378060

[B38] Suzzi G. Gardini F. (2003). Biogenic amines in dry fermented sausages: a review. *Int. J. Food Microbiol.* 88 41–54 10.116/S0168-1605(03)00080-1 14527784

[B39] Tabanelli G. Torriani S. Rossi F. Rizzotti L. Gardini F. (2012). Effect of chemico-physical parameters on the histidine decarboxylase (HdcA) enzymatic activity in *Streptococcus thermophilus* PRI60. *J. Food Sci.* 77 M231–M237 10.1111/j.1750-3841.2012.02628.x 22429258

[B40] Torriani S. Gatto V. Sembeni S. Tofalo R. Suzzi G. Belletti N. (2008). Rapid detection and quantification of tyrosine decarboxylase gene (tdc) and its expression in gram-positive bacteria associated with fermented foods using PCR-based methods. *J. Food Protect.* 71 90–101. 10.4315/0362-028x-71.1.93 18236668

[B41] Zwietering M. H. Jongenburger I. Rombouts F. M. van’t Riet K. (1990). Modelling of the bacterial growth curve. *Appl. Environ. Microb.* 56 1875–1881. 10.1128/aem.56.6.1875-1881.1990 PMC184525 16348228

